# Exsudative Diathesis—A Review*This review has been published in abridged form in the American Journal of Diseases of Children, October, 1915, Vol. X.

**Published:** 1916-05

**Authors:** Carl G. Leo-Wolf

**Affiliations:** Buffalo, N. Y.


### Exsudative Diathesis—A Review*

By CARL G. LEO-WOLF, M. D., of Buffalo, N. Y.

**Historical:** His (33) and Krehl (42). The idea of the diatheses is very old. It played a prominent role in the medicine of ancient Greece, especially the school of Galenus. He considered diathesis and constitution to be the determining factors in the symptoms of disease, and he regarded a faulty mixture of the elements and the temperaments, and the consequent disturbance of the equilibrium of the body as the cause of disease; while he viewed the changes in the organs as a secondary matter only.

This ancient theory of the crases persisted up to the beginning of the modern scientific study of the natural phenomena, when exact examination took the place of philosophical speculation.

Virchow's cellular pathology and the wide-spread use of the microscope finally dislodged the constitutional theory, supplanting it by the pathology of the organs and the principle of organic localization.

Some German clinicians however, such as Henle and Wunderlich, still adhered to the idea of the constitutions, and the French school of medicine, under the leadership of such men as Teissier, Bazin and Lancereaux, kept up the theory of constitution and diathesis.

The German school of medicine of the beginning of this century was forced to acknowledge that the physiological, anatomical-localistic, and etiological considerations of the diseased state did not comprise the sum-total of the morbid processes. They realized that the medical student is still forced to employ, not only the exact results of chemistry, physics and experimentation, but also experience and empirics, which latter are frequently nothing more than a series of obscure impressions. Thus we have come to the conclusion, that the road to truth in medicine leads over careful observation, both at the bedside and in the laboratory, and that we must not be dominated by a skeptic negation of everything savoring of the theoretical.

Medical writers in Europe, especially in Germany and France, have given ample expression to the theories of disposition and constitution. In Pediatrics as well as Dermatology these theories have helped us to understand many a clinical picture which would otherwise have remained veiled.

**The Diatheses:** The term "diathesis" attempts to give ex-

---

*This review has been published in abridged form in the American Journal of Diseases of Children, October, 1915, Vol. X.

pression to the internal relations of heterogenous groups of diseases, due to individual conditions, which are usually congenital, often hereditary. In these physiological stimuli cause an abnormal reaction and morbid phenomena under normal conditions of life.

At present we are able only to recognize certain groups of symptoms, to which we give the name of a diathesis; such as arthritism, lithemia, exsudative diathesis, spasmophilia, status thymico-lymphaticus, mongolism, infantilism, eosinophilia, some neuropathies, hemophilia, perhaps also epilepsy, chlorosis and the hypoplasies of the aortic system. His (33).

The French School of medicine recognizes the "diathése arthritique." This they regard as a disease of the intellectual classes, of those with sedentary habits, and therefore a disease of degenerating races.

Comby (18) considers arthritism as a morbid temperament, a diathesis, which is dormant in childhood and does not appear until adult life, when it will break out in one of its paroxysms of gout, asthma, glycosuria, nephritic colic, migraine, etc. In childhood however the symptoms of the hereditary taint will be observed under the guise of imperfect nutrition, incomplete development, hypertrophies or hyperplasies, a defective arterial system, and an abnormal and easily vulnerable connective tissue. He quotes H. Cazalis, who stated, that the irritability and degeneration of the skin, the mucous membranes, the entire connective tissue, seem in arthritics to be due to the lack of a substance which is easily used up.

Comby thinks that arthritism is mostly inherited from the father, rarely from the mother, also that it is most frequent amongst the urban middle class population, especially Hebrews, people who work mostly with their brains and are overfed.

In well-cared-for nursing babies certain evanescent cutaneous manifestations will be found as a sign of the morbid constitution, such as eczematous patches on the face, prurigo, tenacious and recurring eczema of the cheeks, forehead or scalp, these may also become generalized, they are very difficult to treat, recur constantly and may torment the child for years. Other babies may remain without these manifestations from the skin or mucous membranes, but they will be nervous and excitable, with an ugly disposition and prolonged crying spells, will be poor sleepers and suffer from night terrors, and have spasmophilic symptoms. Others again will have arthritic attacks of headaches, migraine, "cephalalgies uricémiques." pseudo-intermittent fever and arthritic fever.

The so-called paraarthritic signs of the obese arthritic children, according to Comby, are pallor, lymphatism or scrophulosis, exaggerated or irregular appetite, bulimia, indigestion from overeating, habitual constipation or diarrhoea alternating with constipation, gas, borborygmata, distended abdomen, signs of gastric dilatation, painful intestinal colic, muco-membranous enteritis, dysenteriform colitis, cyclic vomiting, hemorrhoids; discharges from the mucous membranes such as conjunctivitis, spasmodic coryza, epistaxis; abundant sweating. These children are very sensitive to drafts, have frequent colds, spasmodic cough with pharyngitis, laryngitis, laryngo-tracheitis; cerebral affections; pains in bones or joints, lassitude in the limbs; persistant headaches, apathy, discouragement, neurasthenia, lack of inclination to either mental or physical exercise; predisposition to chilblains, urticaria, purpura, acne; in girls at puberty chlorosis; spasms of the bladder, seminal emissions, neuralgias, subcutaneous edemata, and priapism even in infants.

Later in life we will observe premature baldness, articular swellings, hydrarthrosis, cracking in the joints and arthritis sicca.

This description of Comby's is rather comprehensive, and it includes, as we will see later, exsudative diathesis as well as all the different diatheses of other writers.

Hutinel (35), another leader of French pediatrics, states that it is, at present at least, very difficult to define arthritism. It is not a disease, but a morbid temperament, a peculiar form of metabolism which may be, in some cases, acquired, but is mainly inherited, and it is a difficult matter to give an exact formula of the special disturbance of metabolism which exists in a given case.

He also describes under arthritism all the different diatheses, and goes as far, as to say, that there seems to be an antagonism between arthritism and tuberculosis, so that tuberculosis will take a slower course in arthritic subjects, with a tendency towards sclerosis and an ultimate cure, although this has lately been denied by other French writers such as Poncet et Leriche, and Léon Bernard.

Hutinel also recognizes as ''état lymphatique,'' the status thymico-lymphaticus of Escherich and Paltauf, which he has observed in children with hereditary syphilis, arthritism, lymphatism and defective nutrition. This however may also be due to overfeeding, badly regulated feeding and poor digestion. He compares these pale and puffy children very aptly with plants, which have been watered too much and which are therefore too juicy. The weight of these children gives a wrong impression of their actual condition, they have little resistance to diseases, they may die suddenly and unexpectedly.

De Monchy (61) thinks that arthritic and exsudative diathesis should be clearly separated, even more so than has been done by Czerny, he considers Stoeltzner's "oxypathy" as very similar to the diathése arthritique."

Galup (24) brings arthritism as a diathesis in relation to anaphylactic phenomena. He states that, not only are lymphatics often the descendants of arthritics, but that they frequently will devolop arthritism later in life.

In English and American medicine we find as lithemia the symptoms which French writers collect under arthritism, Rachford (71 & 72). Others, as for instance Whitmore (95), describe under lymphatism the status thymico-lymphaticus, or they recognize this latter condition only, as for instance Holt (34).

The majority of writers in the English language however fail to mention the diatheses altogether. This neglect is now beginning to be recognized by such men as Cameron (11), who deplores this fact and shows the importance of the diatheses on infant feeding and infant mortality.

Dutch literature is also rather wanting on the subject of the diatheses. Some, like Sthemann (90), refuse to recognize the revival of the ancient theory of the crases, whilst others like Gorter (26), take a standpoint somewhat between the French and German schools.

Ever since the memorable paper of Czerny (12), ten years ago, in which he first mentioned the name of "exsudative diathesis," a great many monographs have been published on the subject of the diatheses, and their importance in medicine has, by some at least, been fully recognized.

Some German authors, like Pfaundler (67), consider it a mistake to confound diathesis with dyscrasia, and the symptoms of a diathesis with the diathesis itself. He considers the special dispositions as independent, and he claims that we can observe many different forms of their combination and coincidence; he recognizes thirty-one free combinations which are without causal connections, and he thinks that isolated predispositions are rare.

v. Behring (4) confines the term diathesis to only a small part of the disposition. He considers a diathesis as an individual, congenial, often inherited condition, in which physiological stimuli cause an abnormal reaction and in which conditions of life, which are safely born by the majority of individuals, produce pathological conditions.

He makes the following divisions of the dispositions:

A. Idiopathic disposition (congenital, constitutional, histopathic or organopathic);

I. Idiosyncrasies (Congenital hypersensitiveness against agents of a definite type, which are normally innocuous);

II. Diatheses (congenital hypersensitiveness against agents of the most diverse types, which are normally innocuous).

B. Toxopathic disposition (isopathic, acquired);

I. Anaphylaxes (isopathically acquired, humorigenous hypersensitiveness towards more or less innocuous agents of a definite type, which may be transmitted from the anaphylactic individual upon normal individuals through the anaphylactic antibodies dissolved in the blood);

II. Nonanaphylactic hypersensitiveness towards toxins (isopathically acquired sensitiveness towards poisons which are either histogenous or cytogenous and which cannot be transferred).

Klotz (40) makes the following divisions:

Eugenetic trophostabile individuals;

I. Congenitally normal infants who thrive on any food.

II. Infants with exsudative diathesis.

Dysgenetic tropholabile individuals;

III. Rachitic infants.

IV. Neuropathic or psychopathic infants.

V. Hypotrophic or hypertrophic infants.

Escherich, Heubner, Moro and others consider the status lymphaticus or lymphatism as a separate constitutional abnormality, whilst Klotz together with Czerny, Salge and others consider it only an extreme type of exsudative diathesis.

Sittler (85) states that exsudative diathesis is found so frequently in children nowadays that one might feel inclined to regard these symptoms merely as the physiological reaction to the unhygienic mode of living and the poor feeding now prevailing; still exsudative diathesis may appear only in some of the children out of a number who are living under the same conditions. According to him the diathesis is inherited from the mother principally.

Pfaundler (65) considers the diatheses of childhood, namely exsudative diathesis of Czerny, status thymico-lymphaticus of Paltauf, infantile arthritismus of Comby, to represent identical conditions, viewed only from different standpoints. He maintains that it is important to separate the cases according to the manifestations they show, whilst he considers the disturbances of the vegetative innervation and the general neuropathic symptoms as only loosely connected with the diathesis.

According to his hypothesis the seat of a disease is to be found in the affected tissues themselves, and the affected organs all come from the mesenchyma. The symptoms may appear in all kinds of combinations, and the causal connection of single symptoms may be doubted. Most likely the different diatheses may be divided into partial dispositions which are truly inherited. At the bottom of these we will then find a functional inferiority of different organs of cell-systems, these can then, by functional tests, be shown to exist during their latency, as for instance a provocation of exsudative symptoms by overfeeding, or Moro's vasomotor test, which consists in a traumatic reaction to a dry injury with the v. Pirquet scarifyer.

**The Etiology of Exsudative Diathesis:** Friedjung (23) is a strong believer in the theory of the organic inferiority as propounded first by Adler, who claimed that heredity does not consist in the transmission of a certain disease, but of one or more inferior organs. Constitutional differences are then the reason why infants may fail to thrive under the best of care, even with well-regulated breast-feeding, also why vastly different methods of artificial feeding may give either good or bad results in the hands of the same investigator.

Lederer (47) finds the cause of exsudative diathesis in the water-metabolism. He does not however find the water in the blood increased, but the retention of water in the tissues is very loose in exsudative diathesis, the absorbtion of water in these children is very rapid in proportion to their manifest symptoms, and this is due to faulty feeding. When these children are then suddenly placed on a sparse diet they will loose the superfluous water within a few days.

Steinitz & Weigert (89) have made repeated metabolism experiments on two infants and they have found a decreased nitrogen and fat metabolism. In their cases the weight-curve remained flat in spite of a sufficient supply of breast-milk and it only attained the normal under "allaitement mixte."

Riesel (85) found that out of thirty-five children whose bodily conditions were marked on their history-charts as fat or pastous, twenty-six had most of the symptoms of exsudative diathesis. These children were mostly brought to him because they were difficult to feed. At first they had usually been in poor condition, with bad colour, reduced panniculus adiposus and turgor. In the second half of their first year however they had gained more than normal in weight, they were shorter and broader than would have corresponded to their age, and they had not learned to walk until they were over eighteen months old. After the third year of life these children usually would be slim again. He also states that atrophy in early infancy and adipositas in late infancy are both due to exsudative diathesis, and they are observed in the same children.

Maillet (53) considers the subcutaneous tissue to be of the greatest importance for the nutrition as well as the defense of the body. Hypertrophy or atrophy of its anatomical elements will disturb this function, paralleling the general condition of obesity, lymphatism or cachexia. These in turn influence the constitution, by weakening the defensive action of the subcutaneous tissue, so that lymphatism aids in localizing a given infection, whilst cachexia and atrophy favors its generalization in the body.

Mendelsohn (57) is an adherent of Bouchard's (9) theory of the retardation of metabolism and of humeral hypoacidity, from which all organs and tissues are in a state of functional insufficiency (eiopragia), in which they are oscillating between sickness and health. This then constitutes a disturbance of the equilibrium between assimilation and dissimilation.

Passler (63) has a rather remarkable though not convincing view of the etiology of exsudative diathesis. He finds this, as well as other related diatheses, to be always caused by chronic streptococcus infections of the buccal cavity, especially caries dentium and pyorrhoea. He claims that thorough cleansing of the mouth will have good results and will even cure eczema.

(To be Continued in June Issue.)

---

**Salvarsan Substitute.** Owing partly to the war, J. E. R. McDonagh has developed "Intramine," di-ortho-amino-thio benzene. The graphic formula consists of two benzene rings, one connected with H2N (?), the other with NH2, and both, next to these combinations, with one valence of sulphur, the other sulphur valences being mutually united. He lays down the general principle that in acute diseases, oxidizing agents should be used first, reducing agents later, the order being reversed in chronic conditions. Metallic compounds generally act as oxidizing agents, non-metallic, like intramine, as reducing agents. He considers the spirochaeta pallida as merely the male phase of a protozoon with a complicated life cycle and to which he applies the name, leucocytozoon syphilidis. (Practitioner, March, 1916).

---

**Ptomain Poisoning From Creamed Codfish.** M. A. Blankenhorn, G. E. Harmon & Paul J. Hanzlik, Cleve. Med. Jour., Feb. 1916. About 80 patients and a number of helpers in Lakeside Hospital, were involved, all recovering, within 12-24 hours, some spontaneously, some after lavage. Bacillus coli communior, (no gas production), other saprophytes and some staphylococci were found, none strictly anerobic. The gastrointestinal symptoms were not directly attributed to infection by the microorganisms but to a base, isolated and producing stimulation of intestine, and uterus and fall of blood pressure. This base was considered a diamine, analogous to putrescine, cadaverine and histamin.

